# Unique Gut Microbiome in HIV Patients on Antiretroviral Therapy (ART) Suggests Association with Chronic Inflammation

**DOI:** 10.1128/spectrum.00708-21

**Published:** 2021-08-11

**Authors:** Aya Ishizaka, Michiko Koga, Taketoshi Mizutani, Prince Kofi Parbie, Diki Prawisuda, Nozomi Yusa, Ayako Sedohara, Tadashi Kikuchi, Kazuhiko Ikeuchi, Eisuke Adachi, Tomohiko Koibuchi, Yoichi Furukawa, Arinobu Tojo, Seiya Imoto, Yutaka Suzuki, Takeya Tsutsumi, Hiroshi Kiyono, Tetsuro Matano, Hiroshi Yotsuyanagi

**Affiliations:** a Division of Infectious Diseases, Advanced Clinical Research Center, the Institute of Medical Science, The University of Tokyo, Tokyo, Japan; b International Research and Development Center for Mucosal Vaccines, the Institute of Medical Science, The University of Tokyo, Tokyo, Japan; c AIDS Research Center, National Institute of Infectious Diseases, Tokyo, Japan; d Department of Applied Genomics, Research Hospital, Institute of Medical Science, The University of Tokyo, Tokyo, Japan; e Department of Infectious Diseases and Applied Immunology, IMSUT Hospital of Institute of Medical Science, The University of Tokyo, Tokyo, Japan; f Department of Laboratory Medicine, Research Hospital, Institute of Medical Science, The University of Tokyo, Tokyo, Japan; g Division of Health Medical Data Science, Health Intelligence Center, the Institute of Medical Science, The University of Tokyo, Tokyo, Japan; h Department of Computational Biology and Medical Sciences, Graduate School of Frontier Sciences, The University of Tokyo, Chiba, Japan; i Department of AIDS Vaccine Development, IMSUT Hospital, The Institute of Medical Science, The University of Tokyo, Tokyo, Japan; Karolinska Institutet

**Keywords:** HIV, microbiome, microbiota, dysbiosis, inflammation, human immunodeficiency virus

## Abstract

Chronic inflammation is a hallmark of human immunodeficiency virus (HIV) infection and a risk factor for the development and progression of age-related comorbidities. Although HIV-associated gut dysbiosis has been suggested to be involved in sustained chronic inflammation, there remains a limited understanding of the association between gut dysbiosis and chronic inflammation during HIV infection. Here, we investigated compositional changes in the gut microbiome and its role in chronic inflammation in patients infected with HIV. We observed that the gut microbiomes of patients with low CD4 counts had reduced alpha diversity compared to those in uninfected controls. Following CD4 recovery, alpha diversity was restored, but intergroup dissimilarity of bacterial composition remained unchanged between patients and uninfected controls. Patients with HIV had higher abundance of the classes *Negativicutes*, *Bacilli*, and *Coriobacteriia*, as well as depletion of the class *Clostridia*. These relative abundances positively correlated with inflammatory cytokines and negatively correlated with anti-inflammatory cytokines. We found that gut dysbiosis accompanying HIV infection was characterized by a depletion of obligate anaerobic *Clostridia* and enrichment of facultative anaerobic bacteria, reflecting increased intestinal oxygen levels and intestinal permeability. Furthermore, it is likely that HIV-associated dysbiosis shifts the immunological balance toward inflammatory Th1 responses and encourages proinflammatory cytokine production. Our results suggest that gut dysbiosis contributes to sustaining chronic inflammation in patients with HIV infection despite effective antiretroviral therapy and that correcting gut dysbiosis will be effective in improving long-term outcomes in patients.

**IMPORTANCE** Chronic inflammation is a hallmark of HIV infection and is associated with the development and progression of age-related comorbidities. Although the gastrointestinal tract is a major site of HIV replication and CD4^+^ T-cell depletion, the role of HIV-associated imbalance of gut microbiome in chronic inflammation is unclear. Here, we aimed to understand the causal relationship between abnormalities in the gut microbiome and chronic inflammation in patients with HIV. Our results suggest HIV-associated gut dysbiosis presents a more aerobic environment than that of healthy individuals, despite prolonged viral suppression. This dysbiosis likely results from a sustained increase in intestinal permeability, which supports sustained bacterial translocation in HIV patients, despite effective therapy. Additionally, we observed that several bacterial taxa enriched in HIV patients were associated with increased expression of inflammatory cytokines. Collectively, these results suggest that gut dysbiosis plays an important role in chronic inflammation in HIV patients.

## INTRODUCTION

The current treatment for human immunodeficiency virus (HIV) infection is antiretroviral therapy (ART), which efficiently suppresses plasma viremia below detectable levels and improves patients’ life expectancy (1). However, the virus is not completely eradicated, with residual viral replication occurring in lymphoid tissues. Numerous studies, including ones from our group, have reported an association of persistent inflammation with viral replication and/or transactivation of residual virus (2, 3). Patients infected with HIV have increased immune activation and chronic inflammation, even during successful ART. This inflammation potentially contributes to the development and progression of multiple diseases, including some cancers, cardiovascular disease, and chronic kidney disease. Patients with HIV are at high risk of these comorbidities compared to age-matched uninfected populations; therefore, HIV infection is increasingly viewed as a chronic disease that enhances aging.

Chronic activation of the immune system is associated with bacterial translocation during both acute and chronic HIV infection (4–7). Gut-associated lymphoid tissue (GALT) is a major inductive site for mucosal immunity, which plays an essential role in intestinal homeostasis. Harboring the majority of the body’s CD4^+^ T lymphocytes, GALT is a major target of HIV infection and a reservoir for viral persistence (8–10). Among the mucosal CD4 T-cell subsets, T helper 17 (Th17) cells, which express high levels of CCR5, the coreceptor for HIV, are preferentially depleted during early HIV infection (11, 12). This depletion results in decreased interleukin-17 (IL-17) secretion (13, 14), which is essential for maintaining gut barrier integrity by increasing expression of tight-junction proteins, including claudin-1 and claudin-2 (15). GALT dysfunction promotes translocation of gut bacteria and microbial products from the gut lumen into the systemic circulation (13, 16). Intestinal permeability, measured by plasma levels of lipopolysaccharide (LPS), the major component of the outer membrane of Gram-negative bacteria, was increased in patients with chronic HIV infection and not fully restored by suppressive ART (5). Given that LPS is known as a potent activator of innate immunity and inflammatory responses, this observation suggests that sustained gut damage and microbial translocation contribute to chronic inflammation during HIV infection.

Another important aspect of HIV infection is its impact on the diversity and composition of the gut microbiome. This affects homeostatic mechanisms, including nutrition, metabolism, and host immune regulation, as well as with microbial imbalance (i.e., dysbiosis), which has been linked with multiple diseases, including type 2 diabetes, cardiovascular disease, chronic kidney disease, and obesity (17–20). Previous studies have shown that the gut microbiome profile affects viral transmission and disease progression in HIV/simian immunodeficiency virus infection (21). In addition, it can influence antibody responses to HIV-1 in rhesus macaques (22), suggesting a contribution of the gut microbiome to the host immune response during HIV infection. However, few studies to date have examined the association between alterations of the microbiome and persistent inflammation during chronic HIV infection (23). Here, we examined the gut microbiomes of Japanese patients with treated and untreated HIV-1 infection and present links between microbiome trends and inflammatory status in patients with long-term suppression of viremia.

## RESULTS

### Study populations.

For a cross-sectional comparison of the gut microbiome, 71 patients with HIV infection and 61 age- and sex-matched uninfected controls were enrolled. Among the HIV-positive patients, 53 were men who have sex with men (MSM) and 66 had been treated with ART for more than 16 months. The remaining five patients were ART naive. The characteristics of the study participants are shown in Table 1. For longitudinal analysis of AIDS patients, three patients participated, with fecal samples obtained before and after treatment initiation.

**TABLE 1:** Baseline characteristics of study participants

Characteristic^*a*^	Value for^*b*^:			
	High-CD4 group (*n* = 61)	Medium-CD4 group (*n* = 38)	Low-CD4 group (*n* = 10)	Healthy controls (*n* = 61)
Age	49.6 ± 11.1	51.4 ± 9.72	49.6 ± 10.8	49.7 ± 12.5
No. (%) males	58 (95.1)	36 (94.7)	10 (100)	58 (95.1)
No. (%) of MSM	46 (75.4)	31 (81.6)	7 (70)	
No. (%) with viral loads <50 copies/ml	61 (100)	37 (97.4)	4 (40)	
CD4 count (cells/μl)	690.0 ± 145.2	405.1 ± 71.4	132.7 ± 76.4	
Nadir CD4 count (cells/μl)	205.1 ± 110.2	116.6 ± 78.2	101.7 ± 66.2	
Time since HIV diagnosis (mo)	139.3 ± 57.5	129.9 ± 65.6	59.2 ± 52.5	
Time on ART (mo)	124.6 ± 62.8	120.2 ± 61.3	74.3 ± 38.9 (*n* = 5; 5 untreated)	
BMI	24.9 ± 4.1	23.7 ± 2.9	22.2 ± 5.2	
No. (%) on ART regimen				
INTSTI	53 (86.9)	35 (92.1)	4 (80)^*c*^	
NRTI	56 (91.8)	35 (92.1)	5 (100)^*c*^	
NNRTI	6 (9.8)	4 (10.5)	0 (0)^*c*^	
PI	6 (9.8)	5 (13.2)	1 (20)^*c*^	

a MSM, men who have sex with men; ART, antiretroviral therapy; BMI, body mass index; INTSTI, integrase strand transfer inhibitor; NRTI, nucleoside reverse transcriptase inhibitor; NNRTI, nonnucleoside reverse transcriptase inhibitor; PI, protease inhibitor.

b Values for continuous variables are means with standard deviations; values for categorical variables are counts and percentages. High CD4, more than 500 cells/μl; medium CD4, between 250 and 500 cells/μl; low CD4, fewer than 250 cells/μl.

c Percentage among patients on ART.

### Fecal microbiome diversity.

**FIG 1:**
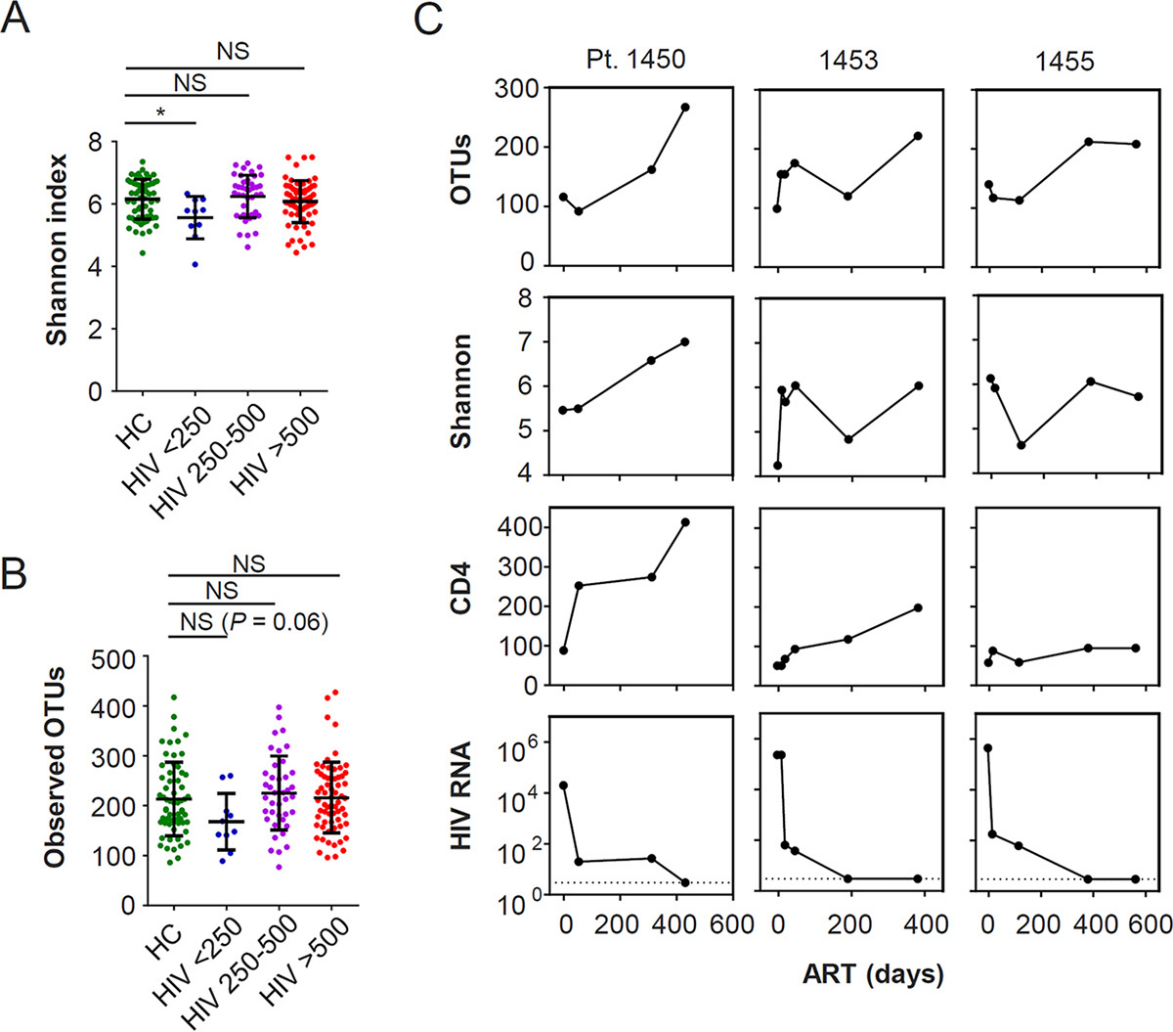
Decreased alpha diversity of gut microbiome is linked to low CD4 counts in HIV patients. (A and B) Comparison of alpha diversity of gut microbiome defined by Shannon index (A) and observed operational taxonomic units (OTUs) (B) among different study groups. (C) Longitudinal changes in alpha diversity, CD4 counts (cells/μl), and plasma HIV RNA (copies/ml) in three AIDS patients. Horizontal dotted lines in graphs of viral load indicate detection limit of plasma viremia (20 copies/ml). The Mann-Whitney U test was used to evaluate the statistical significance of the difference in alpha-diversity in comparison to healthy controls (HC). *, *P < *0.05; NS, not significant.

To clarify the association between the CD4 count and the gut microbiome, we divided patients with HIV into three groups on the basis of CD4 counts: low, fewer than 250 cells/μl; medium, between 250 and 500 cells/μl; and high, more than 500 cells/μl. Rarefaction analysis of operational taxonomic units (OTUs) indicated that sufficient sequencing depth was achieved to avoid biases produced by unequal sample sizes (see Fig. S1 in the supplemental material). In the low-CD4 group, we found decreased alpha diversity (estimated with the Shannon index) relative to uninfected controls (Fig. 1A). Species richness values based on these OTUs were lower than that of controls, although the differences between groups were not statistically significant (*P = *0.06) (Fig. 1B). Alternatively, the medium- and high-CD4 groups exhibited difference neither in the Shannon index nor in OTUs relative to uninfected controls (Fig. 1A and B).

We next compared the longitudinal representation of alpha diversity among three patients with AIDS, all of whom reached viral suppression below detectable levels after treatment initiation (Fig. 1C). All three exhibited restoration of bacterial richness after treatment defined by observed OTUs. For alpha diversity evaluated by the Shannon index, the three patients showed different degrees of restoration: patient 1450, whose CD4 count was 88 cells/μl before and reached 400 cells/μl 2 months after treatment, showed a distinct increase in the Shannon index, whereas patient 1455, who had poor CD4 recovery, showed almost no restoration (Fig. 1C). Patient 1453, whose CD4 recovery was moderate, showed a moderate degree of restoration of the Shannon index (Fig. 1C). These results were consistent with those in Fig. 1A and B, showing that low CD4 counts were more closely linked with lower Shannon indices than with lower numbers of OTUs.

**FIG 2:**
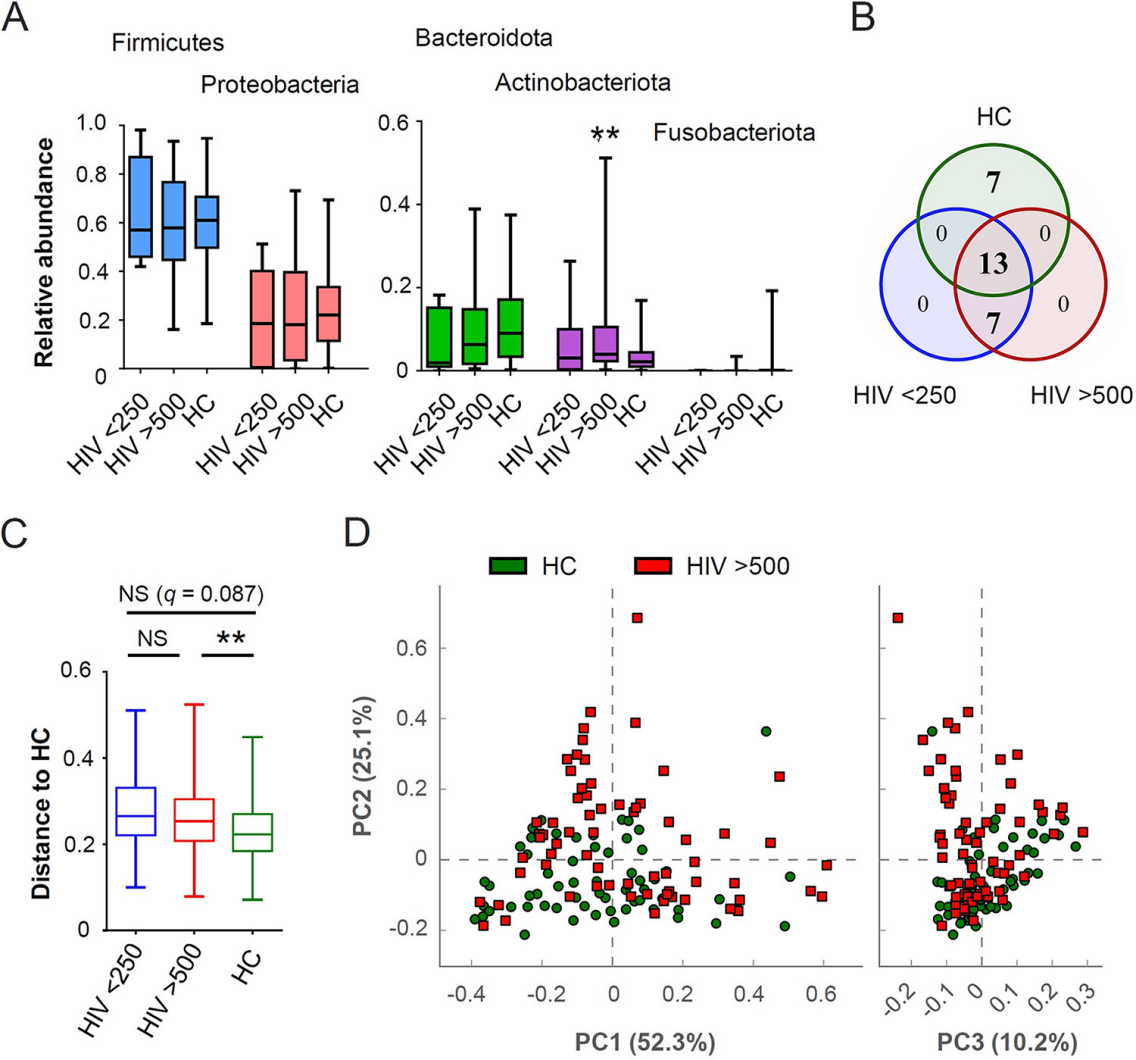
Beta diversity comparisons between HIV patients and healthy controls. (A) Relative abundance of the top five bacteria at the phylum level. Asterisks indicate statistical significance for comparison with healthy controls (HC). (B) Venn diagram of overlaps of the top 20 bacterial genera among groups. (C) Weighted UniFrac distance to healthy controls for each study group. (D) Principal-coordinate (PC) analysis based on weighted UniFrac results between healthy controls and HIV patients with CD4 counts of >500/μl. The Mann-Whitney U test was used to evaluate the statistical significance of the difference in relative abundance in comparison to healthy controls. *, *P < *0.05; **, *P* < 0.01; NS, not significant.

The top five abundant bacterial phyla in individual samples are shown in Fig. 2A. For all subjects, the majority of OTUs represented four phyla: *Firmicutes*, *Proteobacteria*, *Bacteroidetes*, and *Actinobacteria*. We observed a significant increase in the relative abundance of *Actinobacteria* in the high-CD4 group over that in uninfected controls (*P = *0.0031). The Venn diagram in Fig. 2B shows the top 20 bacterial genera from each group. The bacterial taxa in each category are shown in Table 2. The groups of HIV patients with high and low CD4 counts shared all 20 taxa, including 13 that were shared with the uninfected control group. Given that the top abundant taxa were unchanged for both CD4 categories, the microbiome profile of HIV patients with high CD4 counts appeared different from that of uninfected controls, despite the restoration of alpha diversity. In line with this, a permutational multivariate analysis of variance test of beta-diversity, based on weighted UniFrac distance, revealed that bacterial communities were significantly different between the high-CD4 HIV group and uninfected controls (*Q *= 0.006) (Fig. 2C). The intragroup dissimilarity between the two groups was supported by principal-coordinate analysis based on weighted UniFrac results (Fig. 2D). In contrast, the difference between the low-CD4 HIV group and uninfected controls was not statistically significant, which may be due to the small sample size (*n *= 10) in the low-CD4 category (Fig. 2C). Collectively, decreased alpha diversity in HIV patients was linked with low CD4 counts. Following CD4 recovery, alpha diversity was restored; however, bacterial community composition remained different from that of uninfected controls.

**TABLE 2:** Bacteria taxa ranked within the top 20 by abundance in healthy controls and patients with HIV infection

Bacterial taxa^*a*^	Relative abundance (%)^*b*^		
	HC	HIV >500	HIV <250
Ranked within the top 20 in all three groups			
p, *Actinobacteriota*; c, *Actinobacteria*; o, *Bifidobacteriales*; f, *Bifidobacteriaceae*; g, *Bifidobacterium*	2.71	3.58	3.18
p, *Bacteroidota*; c, *Bacteroidia*; o, *Bacteroidales*; f, *Bacteroidaceae*; g, *Bacteroides*	8.40	5.66	3.89
p, *Bacteroidota*; c, *Bacteroidia*; o, *Bacteroidales*; f, *Tannerellaceae*; g, *Parabacteroides*	1.10	0.98	0.88
p, *Firmicutes*; c, *Bacilli*; o, *Lactobacillales*; f, *Streptococcaceae*; g, Streptococcus	1.20	1.37	7.09
p, *Firmicutes*; c, *Clostridia*; o, *Lachnospirales*; f, *Lachnospiraceae*; g, [*Ruminococcus*]_*torques*_group	1.87	1.28	1.85
p, *Firmicutes*; c, *Clostridia*; o, *Lachnospirales*; f, *Lachnospiraceae*; g, *Blautia*	4.19	2.77	2.73
p, *Firmicutes*; c, *Clostridia*; o, *Lachnospirales*; f, *Lachnospiraceae*; g, *Dorea*	2.50	1.57	2.00
p, *Firmicutes*; c, *Clostridia*; o, *Oscillospirales*; f, *Ruminococcaceae*; g, *Faecalibacterium*	14.42	11.29	11.58
p, *Firmicutes*; c, *Clostridia*; o, *Oscillospirales*; f, *Ruminococcaceae*; g, *Subdoligranulum*	8.78	7.43	12.36
p, *Firmicutes*; c, *Negativicutes*; o, *Acidaminococcales*; f, *Acidaminococcaceae*; g, *Phascolarctobacterium*	1.16	2.42	1.18
p, *Firmicutes*; c, *Negativicutes*; o, *Veillonellales*-*Selenomonadales*; f, *Selenomonadaceae*; g, *Megamonas*	2.19	4.52	3.92
p, *Proteobacteria*; c, *Gammaproteobacteria*; o, *Enterobacterales*; f, *Enterobacteriaceae*	6.83	4.45	1.23
p, *Proteobacteria*; c, *Gammaproteobacteria*; o, *Enterobacterales*; f, *Enterobacteriaceae*; g, Escherichia-*Shigella*	14.78	15.83	19.09

Ranked within the top 20 only in HC			
p, *Firmicutes*; c, *Clostridia*; o, *Clostridia*_UCG-014; f, *Clostridia*_UCG-014; g, *Clostridium*_UCG-014	1.41	0.85	0.13
p, *Firmicutes*; c, *Clostridia*; o, *Lachnospirales*; f, *Lachnospiraceae*	1.76	1.66	0.75
p, *Firmicutes*; c, *Clostridia*; o, *Lachnospirales*; f, *Lachnospiraceae*; g, [*Ruminococcus*]_*gnavus*_group	1.35	0.71	0.79
p, *Firmicutes*; c, *Clostridia*; o, *Lachnospirales*; f, *Lachnospiraceae*; g, *Agathobacter*	1.83	2.11	0.13
p, *Firmicutes*; c, *Clostridia*; o, *Oscillospirales*; f, [*Eubacterium*]_*coprostanoligenes*_group; g, [*Eubacterium*]_*coprostanoligenes*_group	1.84	2.16	0.21
p, *Firmicutes*; c, *Clostridia*; o, *Oscillospirales*; f, *Oscillospiraceae*; g, UCG-002	1.19	0.72	0.23
p, *Firmicutes*; c, *Negativicutes*; o, *Veillonellales*-*Selenomonadales*; f, *Veillonellaceae*; g, *Dialister*	1.04	3.66	0.83

Bacterial taxa ranked within the top 20 only in patients with HIV			
p, *Actinobacteriota*; c, *Coriobacteriia*; o, *Coriobacteriales*; f, *Coriobacteriaceae*; g, *Collinsella*	0.33	1.49	1.13
p, *Firmicutes*; c, *Bacilli*; o, *Erysipelotrichales*; f, *Erysipelatoclostridiaceae*; g, *Catenibacterium*	0.50	1.66	3.13
p, *Firmicutes*; c, *Bacilli*; o, *Erysipelotrichales*; f, *Erysipelotrichaceae*; g, *Holdemanella*	0.03	1.50	2.37
p, *Firmicutes*; c, *Bacilli*; o, *Lactobacillales*; f, *Lactobacillaceae*; g, *Lactobacillus*	1.20	0.23	0.87
p, *Firmicutes*; c, *Clostridia*; o, *Lachnospirales*; f, *Lachnospiraceae*; g, *Lachnospira*	0.16	0.18	1.26
p, *Firmicutes*; c, *Clostridia*; o, *Lachnospirales*; f, *Lachnospiraceae*; g, *Roseburia*	0.47	0.37	1.56
p, *Firmicutes*; c, *Negativicutes*; o, *Acidaminococcales*; f, *Acidaminococcaceae*; g, *Acidaminococcus*	0.10	0.22	2.40

a p, phylum; c, class; o, order; f, family; g, genus.

b HC, healthy controls; HIV >500, patients with CD4 counts greater than 500 cells/μl; HIV <250, patients with CD4 counts lower than 250 cells/μl.

### Fecal bacterial composition is altered in patients with HIV.

**FIG 3:**
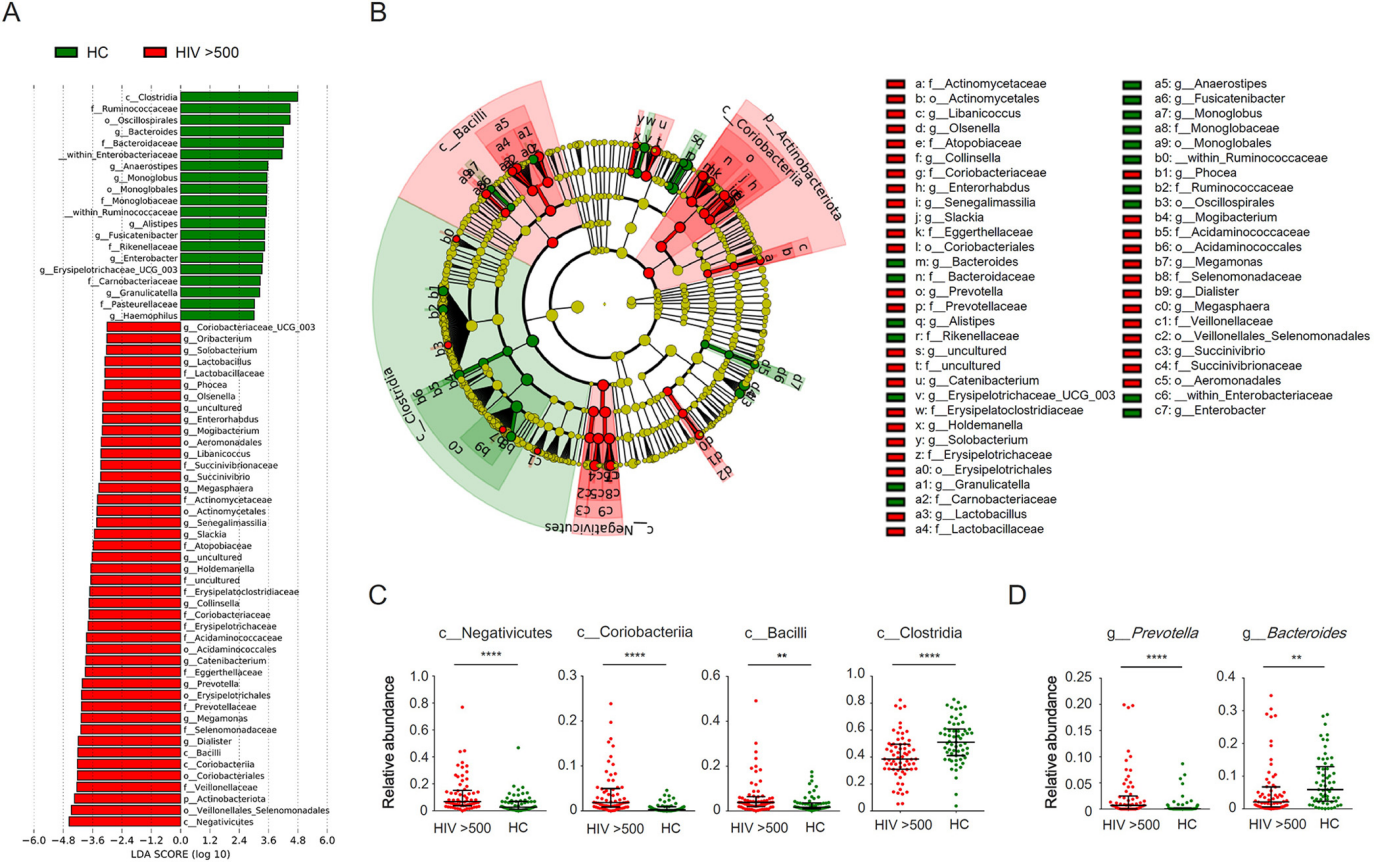
Taxonomic differences between fecal microbiota of HIV patients and healthy controls. (A) Differentially abundant bacterial taxa quantified as linear discriminant analysis effect size (LEfSe) between healthy controls (HC) and HIV patients with CD4 counts higher than 500 cells/μl. Only taxa with an LDA score of >3.0 are shown. (B) Taxonomic cladogram of the data shown in panel A. (C) Relative abundance of the classes *Negativicutes*, *Coriobacteriia*, *Bacilli*, and *Clostridia*. (D) Relative abundance of the genera *Prevotella* and *Bacteroides*. **, *P* < 0.01; ****, *P* < 0.0001.

To identify specific bacterial taxa associated with HIV infection, we compared the bacterial composition between HIV patients with CD4 counts greater than 500 cells/μl and uninfected controls using the linear discriminant analysis (LDA) effect size (LEfSe) algorithm. Thirty-three bacterial taxa were identified to distinguish HIV patients, with a log_10_-transformed LDA score of 3.0 (Fig. 3A). Most of the enriched bacterial populations in HIV patients belonged to the class *Negativicutes*, *Coriobacteriia*, or *Bacilli*, while depleted taxa primarily belonged to *Clostridia* (Fig. 3B and C). At the phylum level, *Actinobacteria* were more abundant in patients with HIV than in uninfected controls, consistent with Fig. 2A. The genera *Collinsella* and *Slackia*, which belong to the class *Coriobacteriia* in this phylum, were enriched in patients from order to genus levels. Among the class *Negativicutes*, *Dialister* and *Megamonas* were enriched from the order to genus levels in HIV patients, as well as *Acidaminococcaceae* from order to family levels. In the class *Bacilli*, *Catenibacterium* and *Holdemanella* were enriched in patients from order to genus levels. In the class *Clostridia*, we observed a depletion of *Ruminococcaceae* in patients from order to family levels as well as *Anaerostipes* from order to genus levels. Finally, HIV patients also exhibited increased abundance of *Prevotella* and depletion of *Bacteroides* from family to genus levels (*P* < 0.001 and *P = *0.022, respectively) (Fig. 3D).

### Functional microbiome profiles are altered in patients with HIV.

**FIG 4:**
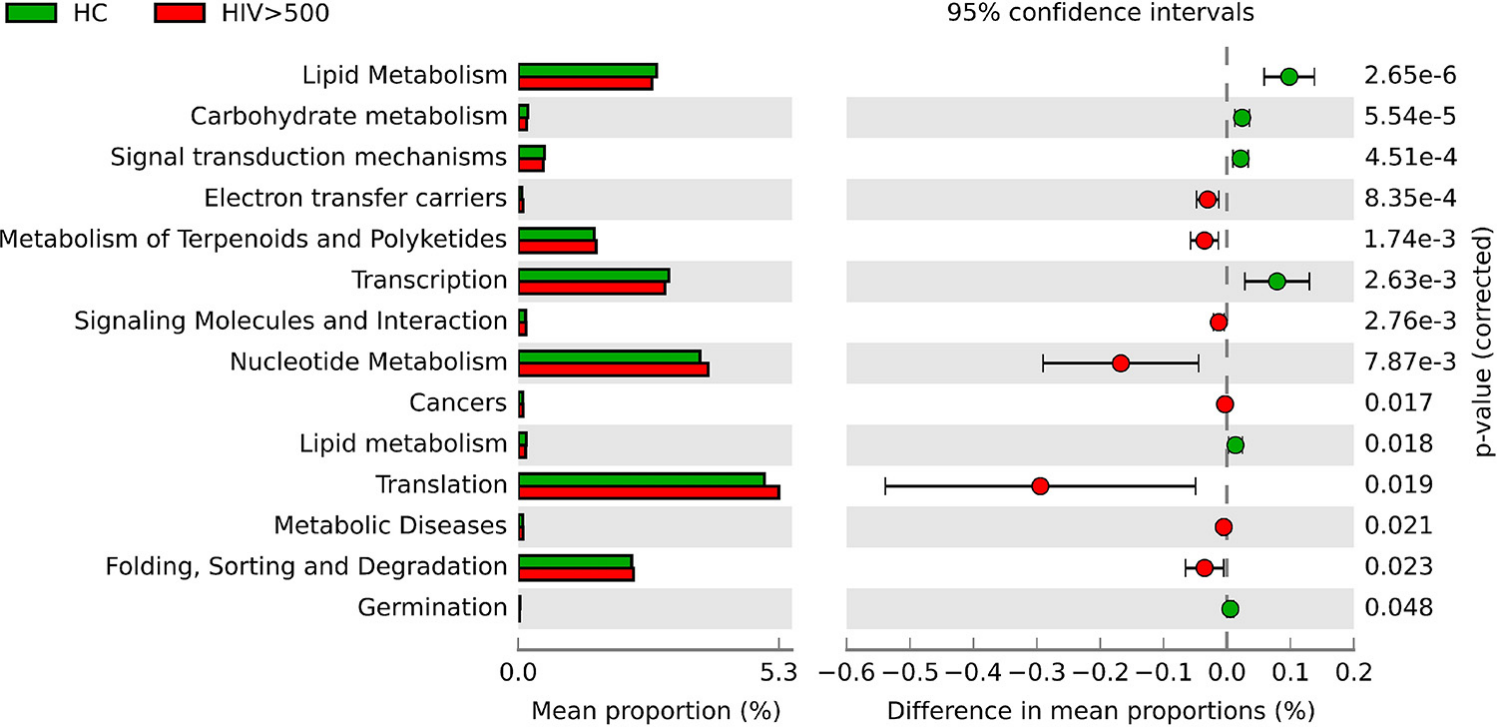
Functional compositions of gut microbiomes in HIV patients predicted by the Kyoto Encyclopedia of Genes and Genomes (KEGG).

To further characterize the HIV-associated gut microbiome, we evaluated functional profiles using the Kyoto Encyclopedia of Genes and Genomes (KEGG) pathway database, which integrates information on intermolecular networks of genes, proteins, metabolism, and signal transduction (24). A significant decrease was found for the lipid metabolism pathway of HIV patients (*P = *2.65 × 10^−6^) (Fig. 4). This evaluation was in line with previous observations of specific microbial associations with obesity; enrichment of *Eggerthella* (class *Coriobacteriia*) and *Holdemanella* (class *Bacilli*) has been associated with elevated blood triglyceride (TG) and decreased high-density-lipoprotein levels (25). Additionally, reduction of the genera *Bacteroides* (class *Bacteroidia*) and *Clostridium* (class *Clostridia*) was associated with decreased body mass index (BMI) and blood TG (25). However, in our analysis the abundance of these bacteria and traits related to obesity were not significantly correlated among HIV patients. Moreover, the differentially abundant taxa between patients with HIV whose BMI was less than 25.0 and uninfected controls were similar to those shown in Fig. 3B (Fig. S2). These observations suggest that gut microbiome changes in chronic HIV patients were not merely derived from obesity, although bacterial taxa related to obesity were enriched.

### Association between gut microbiome and cytokine production.

**FIG 5:**
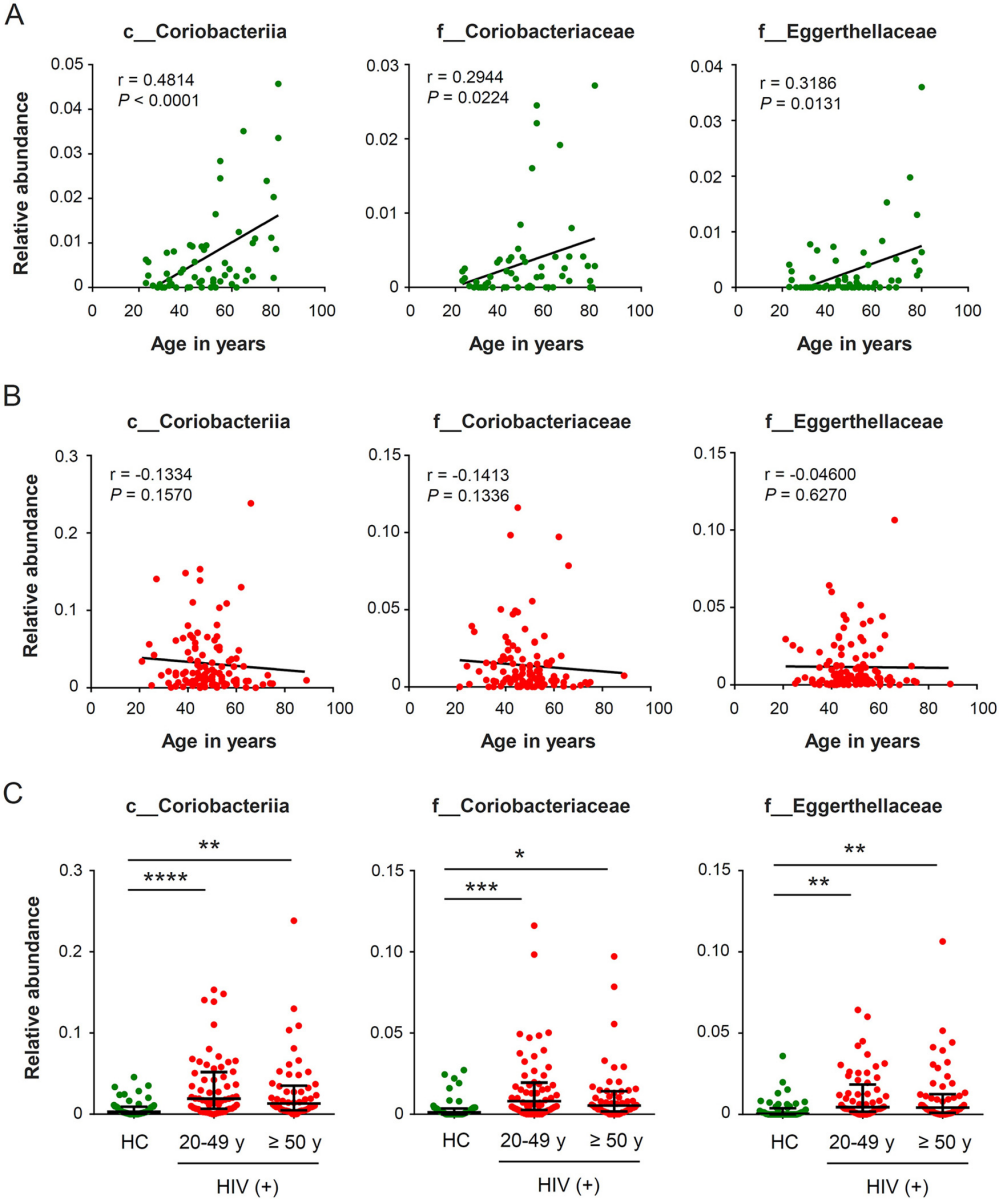
Association between patient age and bacterial taxa of the class *Coriobacteriia*. (A and B) Correlations between age and relative abundance of *Coriobacteriia* in healthy controls (A) and HIV patients (B). (C) Comparison of abundance of *Coriobacteriia* between HIV patients and healthy controls (HC). HIV patients were divided into two groups according to age. *, *P < *0.05, **, *P* < 0.01, ***, *P* < 0.001, ****, *P* < 0.0001.

Several bacterial taxa have been related to age-associated changes in the microbiome. Among the uninfected controls, we found a positive correlation between patient age and abundance of the class *Coriobacteriia*, as well as the families *Coriobacteriaceae* and *Eggerthellaceae* in this class (Fig. 5A). This was consistent with a previous study showing age-related enrichment of these bacterial taxa in cynomolgus macaques during normal, healthy aging (26). However, in HIV patients, no correlation was observed between the abundance of these taxa and patient age (Fig. 5B). When we divided the patients into two groups by age older or younger than 50 years, both groups exhibited elevated abundance of these taxa relative to that in uninfected controls (Fig. 5C). Given that these taxa have also been associated with chronic inflammation in a mouse model of obesity (27), the persistent inflammation observed in HIV patients is likely relevant to microbial composition.

**FIG 6:**
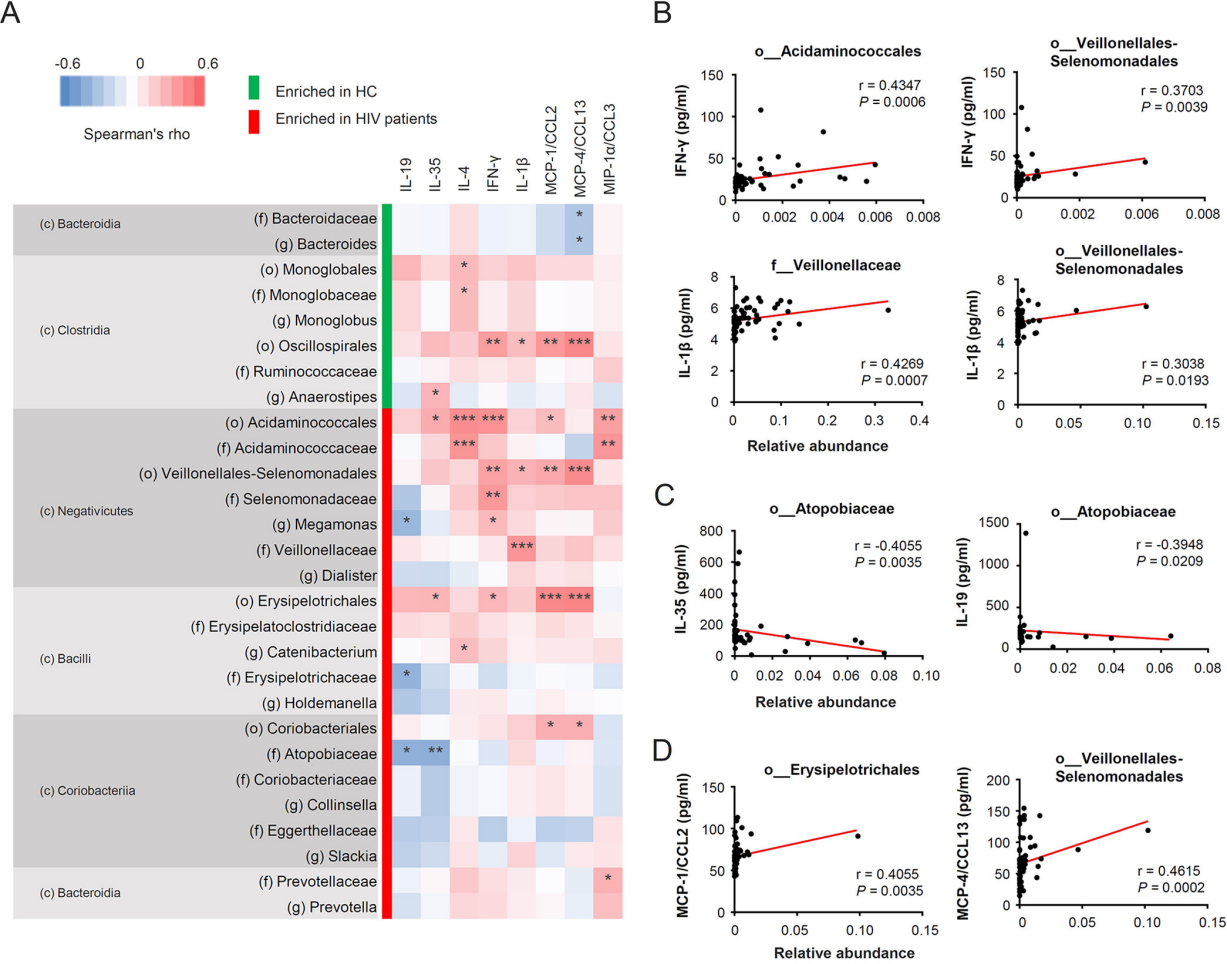
Relationship between bacterial composition and systemic inflammation among HIV patients. (A) Heat map of Spearman’s correlation coefficient between microbial profiles and expression levels of plasma cytokines and chemokines among HIV patients. Bacterial taxa with LDA scores higher than 3.5 in Fig. 3A are shown. *R* values are represented as indicated by the color key. (B to D) Correlations between cytokine expression and relative abundance of individual bacteria among HIV patients. c, class; o, order; f, family; g, genus. *, *P < *0.05, **, *P* < 0.01, ***, *P* < 0.001.

To clarify the associations between the gut microbiome and persistent inflammation in HIV patients, we measured cytokine and chemokine levels in peripheral blood of patients. The overall correlation between cytokine expression and taxa enriched or reduced in HIV patients is shown in Fig. 6A. Among the taxa enriched in HIV patients—*Negativicutes*, *Bacilli*, and *Coriobacteriia*—*Negativicutes* are Gram-negative and possess an outer membrane structure with LPS (28). We found positive correlations between the relative abundance of multiple bacterial taxa in this class and plasma levels of inflammatory gamma interferon (IFN-γ) and IL-1β among patients with HIV (Fig. 6A and B). This is in line with the fact that IFN-γ and IL-1β are representative cytokines that mediate the LPS-induced immune response (29). Furthermore, several taxa enriched in HIV patients, including the families *Erysipelotrichaceae* in *Bacilli* and *Atopobiaceae* in *Coriobacteriia*, negatively correlated with levels of the anti-inflammatory cytokines IL-19 and IL-35 (Fig. 6A and C). Collectively, our results show that changes in the gut microbiome in patients with HIV are associated with inflammation, which is consistent with the bacterial taxa enriched in HIV patients exhibiting positive correlations between some chemokines targeting monocytes, including monocyte chemoattractant protein 1 (MCP-1/CCL2), MCP-4/CCL13, and macrophage inflammatory protein 1α (MIP-1α/CCL3) (Fig. 6A and D).

## DISCUSSION

Our present study showed an association between low CD4 counts and decreased alpha diversity of the gut microbiome in patients with HIV. Following treatment and CD4 recovery, alpha diversity was restored; however, microbiome profiles were different from those of healthy controls. One major difference we observed with HIV infection was a *Prevotella*-rich, *Bacteroides*-poor enterotype (Fig. 3D). As 75.4% of the patients enrolled in this study were men who have sex with men (MSM), this observation was consistent with those from previous studies showing that this enterotype is a hallmark of an MSM-associated gut microbiome, independent of HIV infection (30–32).

Another important alteration of the gut microbial profile we observed in patients with HIV is that it is strongly linked to systemic inflammation during effective ART. Inflammation is caused by excessive activity of the Th1 response (33) and we found that HIV-associated dysbiosis was correlated with Th1-polarized cytokine profiles. LPS from Gram-negative bacteria is known to effectively induce IFN-γ secretion, which has a major effect on promoting Th1 differentiation (34, 35). We found that the level of IFN-γ secretion among HIV patients was primarily associated with an increased abundance of bacterial taxa belonging to the Gram-negative class *Negativicutes*. An increased Th1 response was consistent with our finding that these *Negativicutes* taxa positively correlated with IL-1β and with monocyte-targeting chemokines, including MCP-1, MCP-4, and MIP-1α. Furthermore, IFN-γ inhibits Th17 differentiation, which leads to a reduction in IL-17-mediated expression of tight junction proteins, including claudin-1 and claudin-2 (15). Disruption of epithelial tight junctions results in gut leakage, increasing bacterial translocation, which can further increase IFN-γ and LPS-induced inflammatory responses. Together, our results suggest that the gut microbiomes of patients with HIV are more inflammatory than those of healthy controls.

We found a dysbiotic pattern associated with higher intestinal oxygen concentration. Generally, a healthy intestinal microbiome is largely dominated by obligately anaerobic bacteria whose fermentation increases the production of short-chain fatty acids (SCFAs). One of the major SCFAs is butyrate, which has been shown to maintain the anaerobic environment in the intestine by facilitating oxygen consumption by epithelial cells and improving intestinal barrier function (36). In patients with HIV, we observed a depletion of butyrate-producing bacteria, including the family *Ruminococcaceae* and some genera belonging to the family *Lachnospiraceae*. A reduced genetic potential for butyrate production was supported by KEGG analysis, revealing decreased enrichment of pathways involved in carbohydrate metabolism. Higher intestinal oxygen concentration in patients with HIV was supported by the increased abundance of facultative anaerobic bacteria, including *Lactobacillus*, several members of the family *Erysipelotrichaceae*, and the order *Aeromonadales*. Increased oxygen concentration can further deplete butyrate production, which in turn disrupts gut barrier function and enhances microbial translocation. Enrichment of facultative anaerobic bacteria is in line with a previous study (37) where naive and ART patients were included in a single analysis. In the present study, we observed that this pattern of gut dysbiosis continues even in patients with prolonged viral suppression on ART, suggesting a sustained interaction between gut dysbiosis and chronic inflammation. A similar dysbiotic pattern has been observed with inflammatory bowel disease (IBD), whose clinical features partially overlap those of HIV infection. Both IBD and HIV infection exhibit systemic inflammation along with bacterial translocation, supporting the hypothesis that there is a feed-forward cycle between chronic inflammation and HIV-associated gut dysbiosis.

The role of gut dysbiosis in chronic HIV infection suggests that reducing intestinal inflammation and increasing gut barrier function will be effective in improving the prognosis of patients with HIV. In IBD, differential clinical approaches to correcting gut microbiome imbalance include fecal microbial implantation, dietary interventions, and probiotic/prebiotic supplementation (38–41). These trials have shown mixed results, with some showing therapeutic reductions in inflammation and others showing limited efficacy. However, these observations will be helpful in enabling a better understanding of, and designing new microbiome-based therapies for, HIV infection.

The primary limitation of our study is its lack of HIV-negative MSM controls, making it difficult to determine whether the microbiome changes we observed are due to sexual preference or HIV infection. MSM have microbiomes characterized by an increased abundance of *Prevotella* and depletion of *Bacteroides*, independent of HIV status (30–32). As in other developed countries, MSM are at highest risk of HIV infection in Japan. In the present study, the *Prevotella*-rich enterotype was observed among HIV patients, the majority of whom were MSM, whereas the healthy control men in the general population showed the *Bacteroides*-rich enterotype. Vujkovic-Cvijin et al. reported HIV-associated dysbiosis characterized by enrichment of *Gammaproteobacteria* and depletion of *Lachnospiraceae* and *Ruminococcus*, regardless of sexual preference (31). The HIV-associated microbial profile that they observed was similar to ours in that it was characterized by enrichment of facultative anaerobic bacteria and a decline in butyrate-producing bacteria (31). Given this similarity, it is likely that the microbial shift we observed in HIV patients is driven by HIV infection, rather than the MSM-associated enterotype.

In summary, our study showed that gut dysbiosis associated with HIV infection persists despite successful ART. We observed a marked depletion of the class *Clostridia* and enrichment of different kinds of facultative anaerobes, suggesting sustained increase in intestinal permeability during ART. We also found an increased abundance of *Negativicutes* in HIV patients associated with Th1-polarized cytokine profiles. It is likely, therefore, that HIV-associated dysbiosis shifts the host’s immunological balance toward inflammatory Th1 responses. We speculate that the involvement of gut dysbiosis in chronic inflammation and immune exhaustion needs to be addressed to improve health outcomes for patients with HIV. Further understanding of the mechanism underlying this feed-forward cycle will reduce morbidity associated with inflammation and lead to the development of new therapeutic strategies to produce a functional cure for HIV infection.

## MATERIALS AND METHODS

### Study subjects and sample collection.

This prospective study involved 109 patients with HIV infection and 61 healthy controls (Table 1). Written informed consent was obtained from each participant prior to enrollment. Ethical clearance was obtained from the Institute of Medical Science of the University of Tokyo (reference no. 28-55-0330). Participants who had consumed antibiotics within the previous 2 weeks were excluded. This study was conducted in accordance with the Declaration of Helsinki.

### DNA extraction.

Stool samples were washed three times in SM-plus buffer (100 mM NaCl, 50 mM Tris-HCl [pH 7.4], 8 mM MgSO_4_, 5 mM CaCl_2_, and 0.01% gelatin) and centrifuged. Pellets were resuspended in SM-plus buffer, and large debris was removed by filtering through a 100-μm cell strainer. The resulting suspensions were incubated with 20 mM EDTA, 500 U/ml achromopeptidase (Sigma-Aldrich), and 0.1 mg/ml human lysozyme (Sigma-Aldrich) at 37°C for 1 h, followed by overnight incubation with 50 μg/ml protease K (Nacalai Tesque) and 0.05% sodium dodecyl sulfate at 37°C. Bacterial DNA was extracted using phenol-chloroform and purified with a QIAquick PCR purification kit (Qiagen).

### DNA libraries and sequencing.

The 16S V3 and V4 regions were amplified using the Kapa SYBR Fast qPCR kit (Kapa Biosystems). The primary amplification was initial denaturation at 94°C for 2 min, then 20 cycles at 98°C for 10 s and 68°C for 15 s using forward primer 5′-ACACGACGCTCTTCCGATCTCCTACGGGNGGCWGCAG-3′ and reverse primer 5′-GACGTGTGCTCTTCCGATCTGACTACHVGGGTATCTAATCC-3′. PCR products were purified with Agencourt AMPure XP magnetic beads (Beckman Coulter) per the manufacturer’s instructions. The secondary amplification was initial denaturation at 94°C for 45 s and then 8 cycles of 98°C for 15 s, 50°C for 30 s, and 72°C for 30 s using NEBNext multiplex oligonucleotides for Illumina (dual-index primers, set 1; New England Biolabs [NEB]). These products were purified as described above and verified using agarose gel electrophoresis. Samples were normalized and pooled, followed by sequencing on the MiSeq platform using the MiSeq reagent kit V3 (Illumina).

### Sequence analyses and statistics.

The 16S rRNA reads were analyzed using the Quantitative Insights Into Microbial Ecology version 2 (QIIME2) pipeline, version 2020.6 (42). Paired-end reads were merged and denoised using DADA2 (43). Thereafter, sequences were clustered into OTUs at 97% similarity, and taxonomy was assigned using the Silva reference database, version 138 (44). The OTU table was rarified at an even sampling depth of 10,000 per sample to avoid biases produced by differences in sequencing depth. The linear discriminant analysis (LDA) effect size (LEfSe) algorithm (https://huttenhower.sph.harvard.edu/galaxy/) (45) was used to determine bacterial taxa with significantly different abundances between groups with an LDA score of >3.0 at a *P* value cutoff of <0.05. Phylogenetic Investigation of Communities by Reconstruction of Unobserved States (PICRUSt) version 1.1.1 (http://galaxy.morganlangille.com/) (46) was used to estimate microbial metabolic functions based on the Kyoto Encyclopedia of Genes and Genomes (KEGG) pathway database (24). Data from PICRUSt were analyzed and visualized using the Statistical Analysis of Taxonomic and Functional Profiles software, version 2.1.3 (47). The Mann-Whitney U test was used for comparison of continuous variables between two groups. Spearman's correlation analysis was conducted to identify relationships between fecal microbiome abundance and cytokine expression.

### Cytokine profiling.

Plasma cytokine levels were quantified using a Bio-Plex System (Bio-Rad Laboratories) using two multiplex assay kits, the Bio-Plex Pro human chemokine panel (40-Plex no. 171AK99MR2) and Bio-Plex Pro human inflammation panel 1 (37-Plex no. 171AL001M), in accordance with the manufacturer's specifications.

### Data availability.

Data described in this study are openly available in DNA Data Bank of Japan (DDBJ) (https://ddbj.nig.ac.jp/DRASearch; accession number DRA012374).
