# Supplementary material for: Unique Gut Microbiome in HIV Patients on Antiretroviral Therapy (ART) Suggests Association with Chronic Inflammation
Source: Microbiol Spectr. 2021 Aug 11;9(1):10.1128/spectrum.00708-21. doi: 10.1128/spectrum.00708-21 (PMC8552706; doi:10.1128/spectrum.00708-21)
Supplement: SUPPLEMENTAL FILE 1 — Supplemental material. Download SPECTRUM00708-21_Supp_1_seq10.pdf, PDF file, 0.2 MB [file spectrum00708-21_supp_1_seq10.pdf]

Supplementary Fig. 1

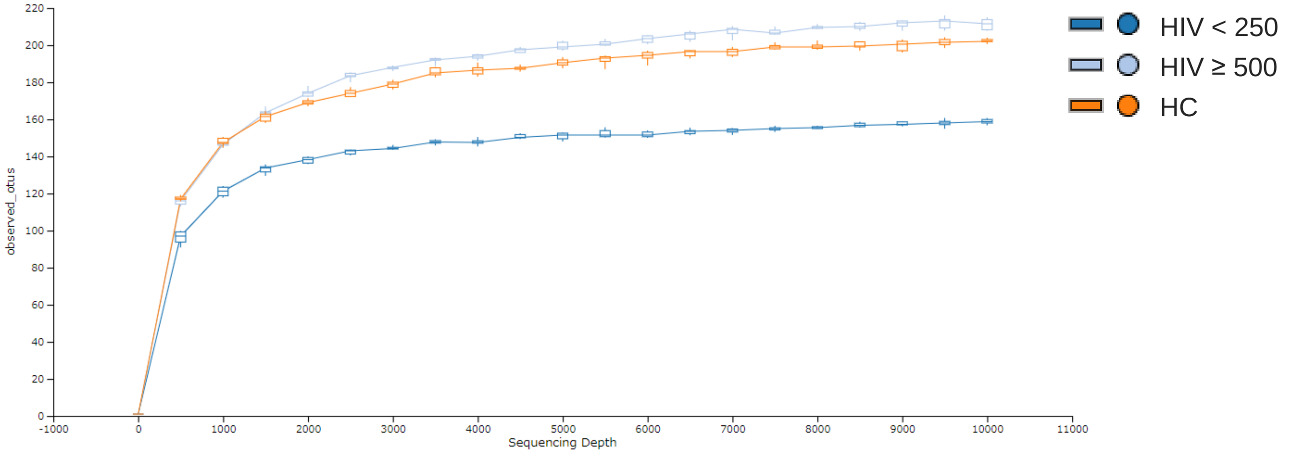

Supplementary Figure 1. Rarefaction analysis based on observed OTUs.

Supplementary Fig. 2

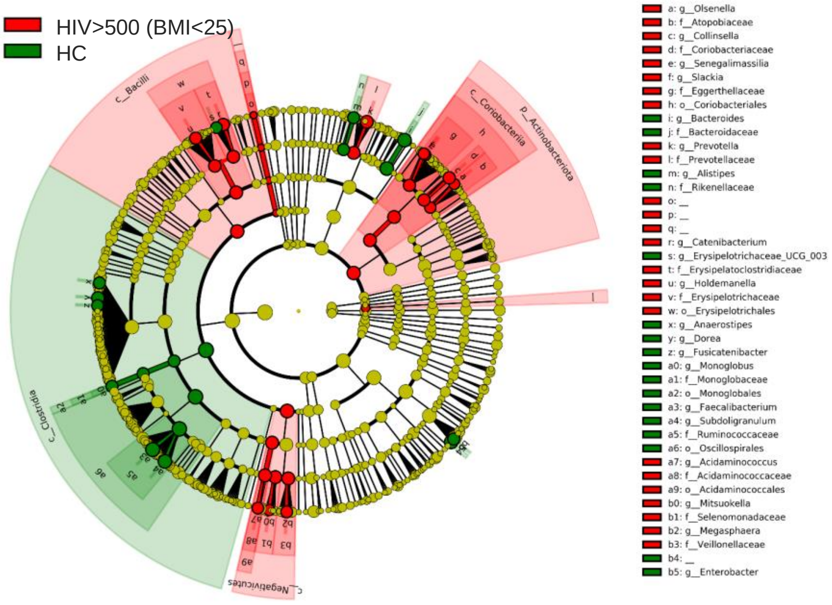

Supplementary Figure 2. Linear discriminant analysis (LDA) effect size (LEfSe) comparison of differentially abundant bacterial taxa between HIV patients whose BMI was lower than 25 and healthy controls.
